# Influence of radiology expertise on the perception of nonmedical images

**DOI:** 10.1117/1.JMI.5.3.031402

**Published:** 2017-12-11

**Authors:** Brendan Kelly, Louise A. Rainford, Mark F. McEntee, Eoin C. Kavanagh

**Affiliations:** a St. Vincent’s University Hospital, Department of Radiology, Elm Park, Dublin, Ireland; b University College Dublin, School of Medicine and Medical Science, Belfield, Dublin, Ireland; c University of Sydney, Medical Radiation Science, Camperdown, New South Wales, Australia; d Mater Misicordiae University Hospital, Radiology, Dublin, Ireland

**Keywords:** image perception, eye tracking, search pattern, diagnostic accuracy, nonradiological images

## Abstract

Identifying if participants with differing diagnostic accuracy and visual search behavior during radiologic tasks also differ in nonradiologic tasks is investigated. Four clinician groups with different radiologic experience were used: a reference expert group of five consultant radiologists, four radiology registrars, five senior house officers, and six interns. Each of the four clinician groups is known to have significantly different performance in the identification of pneumothoraces in chest x-ray. Each of the 20 participants was shown 6 nonradiologic images (3 maps and 3 sets of geometric shapes) and was asked to perform search tasks. Eye movements were recorded with a Tobii TX300 (Tobii Technology, Stockholm, Sweden) eye tracker. Four eye-tracking metrics were analyzed. Variables were compared to identify any differences among the groups. All data were compared by using nonparametric methods of analysis. The average number of targets identified in the maps did not change among groups [mean=5.8 of 6 targets (range 5.6 to 6 p=0.861)]. None of the four eye-tracking metrics investigated varied with experience in either search task (p>0.5). Despite clear differences in radiologic experience, these clinician groups showed no difference in nonradiologic search pattern behavior or skill across complex images. This is another viewpoint adding to the evidence that radiologic image interpretation is a learned skill and is task specific.

## 1 Introduction

Radiologic expertise has been shown to be task specific rather than innate and founded on deliberate practice.^1^^–^^3^ Identifying and codifying the elements of expertise have been the subject of previous work^4^^,^^5^ and indeed attempts have been made to understand it within mathematical^6^ and holistic^7^^,^^8^ frameworks. Eye-tracking metrics, which quantify visual search, correlate consistently with expertise.^4^^,^^7^^,^^9^ The development of visual search from naive to expert participants in the field of pathology has been examined^10^ with many interesting parallels for the field of radiology.

Regarding nonradiologic images, it has been shown that expert radiologists are no better at complex search tasks^1^ than lay people. Both diagnostic accuracy and visual search behavior have been shown to depend on experience with specific kinds of images.^2^^,^^11^

Previous work by the authors^9^ identified that performance increased with experience and that expert visual search appeared to develop before expert diagnostic accuracy. The authors proposed that visual search and diagnostic accuracy, two separate but linked elements to radiologic expertise, could develop separate from, though not independent of, each other.

### 1.1 Study Objectives and Hypotheses

The objective of this study was to characterize whether physicians with different levels of radiologic expertise perform differently in two specific nonradiologic search tasks that involve target identification.

Our hypothesis was that both visual search skill (measured by eye-tracking metrics) and diagnostic accuracy (measured by target identification) would not be different among the groups.

## 2 Methods

### 2.1 Study Design

This prospective study was granted institutional review board approval with exemption from full ethical review granted for this study on the basis that it did not involve collection or display of patient data and used a set of nonclinical open-access images. This paper was prepared in accordance with the Standards for Reporting Diagnostic Accuracy Studies checklist.^12^

**Fig. 1:**
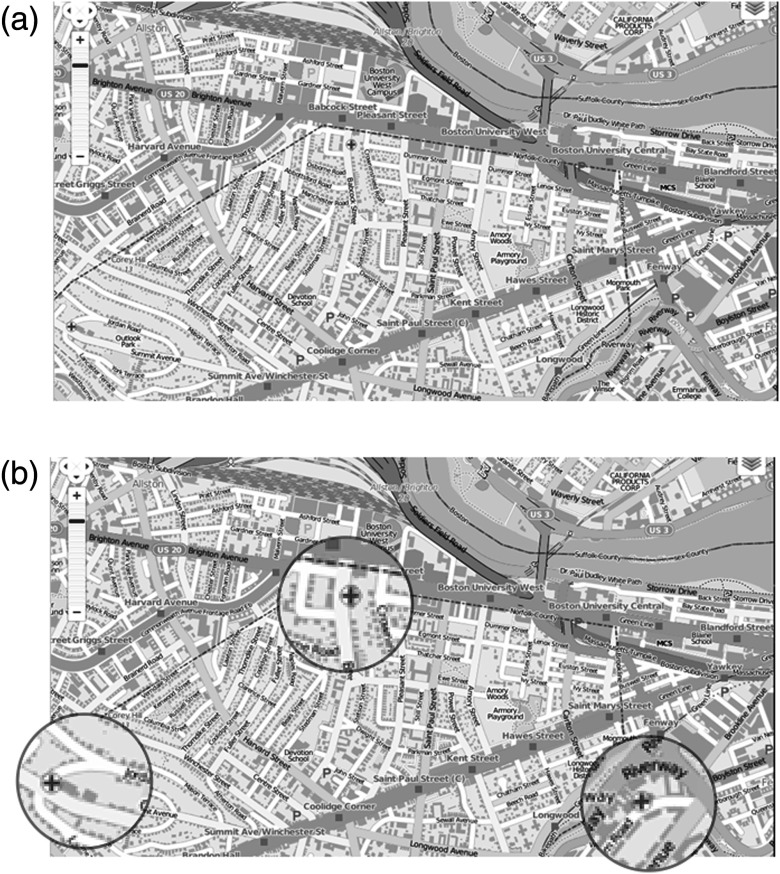
(a) and (b) A map of Boston, one of three maps used in the experiments: (a) the image as it was encountered by the participants and (b) the three hospital targets enlarged.

**Fig. 2:**
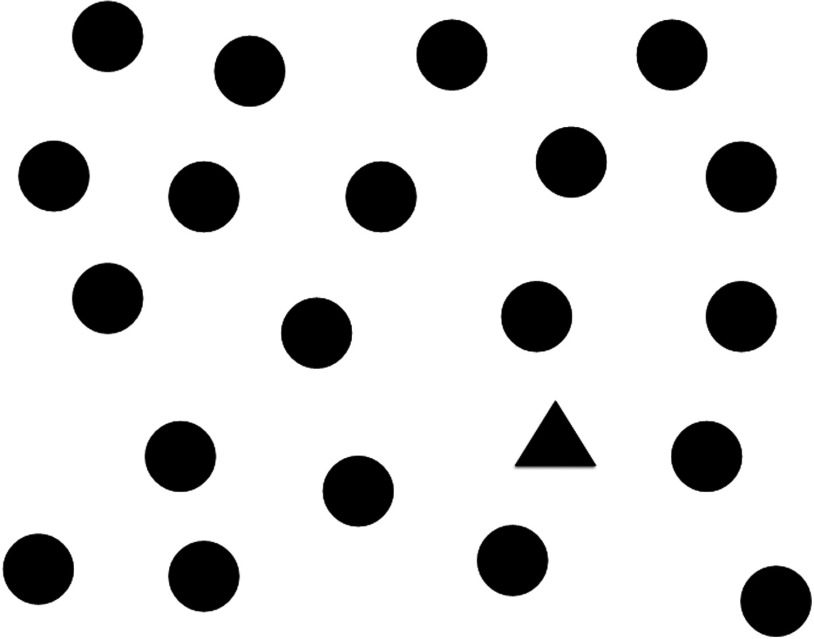
One of three sets of shapes used in the experiments.

This study forms the second half of a two part experiment. In the first half, participants were asked to locate pneumothoraces in chest x-ray (CXR) while having their gaze tracked by a noninvasive eye-tracking device.^9^ Immediately after this experiment, participants began a second experiment in which they were shown three maps [an example is shown in Fig. 1(a)] and were asked to identify all the hospitals, represented by a (+) symbol, [Fig. 1(b)] in that map. The target (+) symbol that designates a hospital was shown to the participants before they began the experiment. They were then presented with three images made up of geometric shapes (an example is shown in Fig. 2) and were asked to move on when they identified a specific shape.

Performance in the initial experiment was estimated by the reference standard in the second experiment (i.e., we are comparing performance in nonradiological search tasks to performance in a specific radiological search task). This was a prospective study.

### 2.2 Participants

Four groups of participants were included in the study. All were medical doctors in one major Irish university affiliated teaching hospital or one peripheral clinical center within the same hospital group. The first group consisted of seven medical interns in their initial week of clinical internship, all of whom completed the MB, BCh, BAO medical degree. Seven of these interns formed a convenience sample from the annual medical rotations, and one was excluded because of poor eye-tracker sample collection (<50%). The second group consisted of five senior house officers (SHOs) (the equivalent to second-year medical residents in the North American system), who completed their intern year in nonradiologic specialties and were part of the medical on-call rotation. The third group consisted of four radiology registrars (radiology residents) in their fourth year of training. The fourth group consisted of five consultant radiologists, with mean clinical experience of 11 years after radiology board certification. Three of the five radiologists specialized in chest imaging, but all were responsible for reporting the same number of chest radiographs annually at a university teaching hospital.

### 2.3 Eye Tracker

The eye movements of participants were tracked with a Tobii TX300 eye tracker (Tobii Technology, Stockholm, Sweden) and an integrated monitor. Display characteristics include an aspect ratio of 16:9 and resolution of 1920×1080  pixels, with a typical screen response time of 5 ms. The eye-tracker sampling rate was 300 Hz. Zooming and panning were not permitted to ensure that eye-tracking data were not influenced by fixations related to these tools rather than the detection task.

### 2.4 Experimental Procedure

Experiments were conducted in a viewing area that was adjacent to but separate from the radiology department. Distractions were minimized and conditions, including monitor display, temperature, and ambient lighting, were maintained in accordance with optimum viewing standards.^13^ The search task was explained to the participants, and they were familiar with the interface from the previous study. The eye tracker was calibrated to each individual’s eye movements at the beginning of the experiment, and a full calibration was carried out at the beginning of each of the four days of experiments.

Before the experiments, “regions of interest” (ROI) were applied to each target within the source images. An acceptance radius of 50 pixels was allowed. A tracked visual fixation within the ROI was deemed as positive identification of the target.

The search task began with a set of instructions (search the image until you are satisfied that you have found all the hospitals along with an image of the hospital icon), and they were presented with three images of maps. Two of the maps contained three targets each, and one map had zero targets. Participants were not told how many hospitals were located on the map. After moving on, they were presented with a multiple choice question (MCQ) asking how many hospitals were identified (giving the options 0, 1, 2, 3, 4, 5, and >5). Results were recorded electronically. Following these images, they were shown another set of instructions (search the image until you have found the “odd shape out” and then progress to the next image) followed by the sets of shapes. Whether they progressed after fixation on the correct shape was recorded electronically. Each participant’s eye movements were tracked as they reviewed the images. One participant was excluded from the analysis, as there were a large number of missing samples.

### 2.5 Analysis

The number of detected targets on the maps was recorded for each participant. Visual search fixations were reviewed to examine if any errors were perceptual (target not fixated) or decision based (target fixated but not recorded). The number of targets correctly identified was deemed to be the measure of diagnostic accuracy. Fixations on the shapes were reviewed to ensure that the correct shape had been identified before proceeding to the next image. Progression to the next image directly after identification of the target shape was deemed to be the measure of diagnostic accuracy.

For the eye-tracking analysis, the following metrics were analyzed: (a) time to first fixation (how many seconds before the participant fixated on the target), (b) fixations before the ROI (how many fixations are made before a fixation was made on the ROI), (c) total fixations, (d) mean fixation time, and (e) maximum visit count (the number of times an ROI was left and revisited). Smaller values for each of these metrics are indicative of better performance. The diagnostic accuracy data (which are the comparison of four means) were analyzed with the Kruskal–Wallis test. Analysis of the eye-tracking metrics was with the Friedman method. p<0.05 was considered to indicate a significant difference.

## 3 Results

The results of the identification of targets on the complex images are shown in Table 1. There was no statistically significant difference in identification of targets in any group using the Kruskal–Wallis test (p=0.861). Intergroup variation was also nonsignificant (p>0.6) All decisions were perception based rather than decision-based errors. This means that the readers did not make a fixation on the target although their gaze may have passed over the target in a saccadic movement. When each group was compared with the experience-adjacent groups (consultant versus registrar, registrar versus SHO, and SHO versus intern), again no statistically significant differences were found. All participants correctly identified the geometric target shape and then progressed on to the next image.

**Table 1:** Number of targets found on maps. There were six targets in total.

Participant	Consultants	Registrar	SHO	Intern
1	6	6	6	5
2	5	6	6	6
3	6	5	6	6
4	6	6	6	6
5	5	—	6	6
6	—	—	—	6
Mean (SD)	5.6 (0.54)	5.75 (0.5)	6 (0.0)	5.83 (.40)

**Fig. 3:**
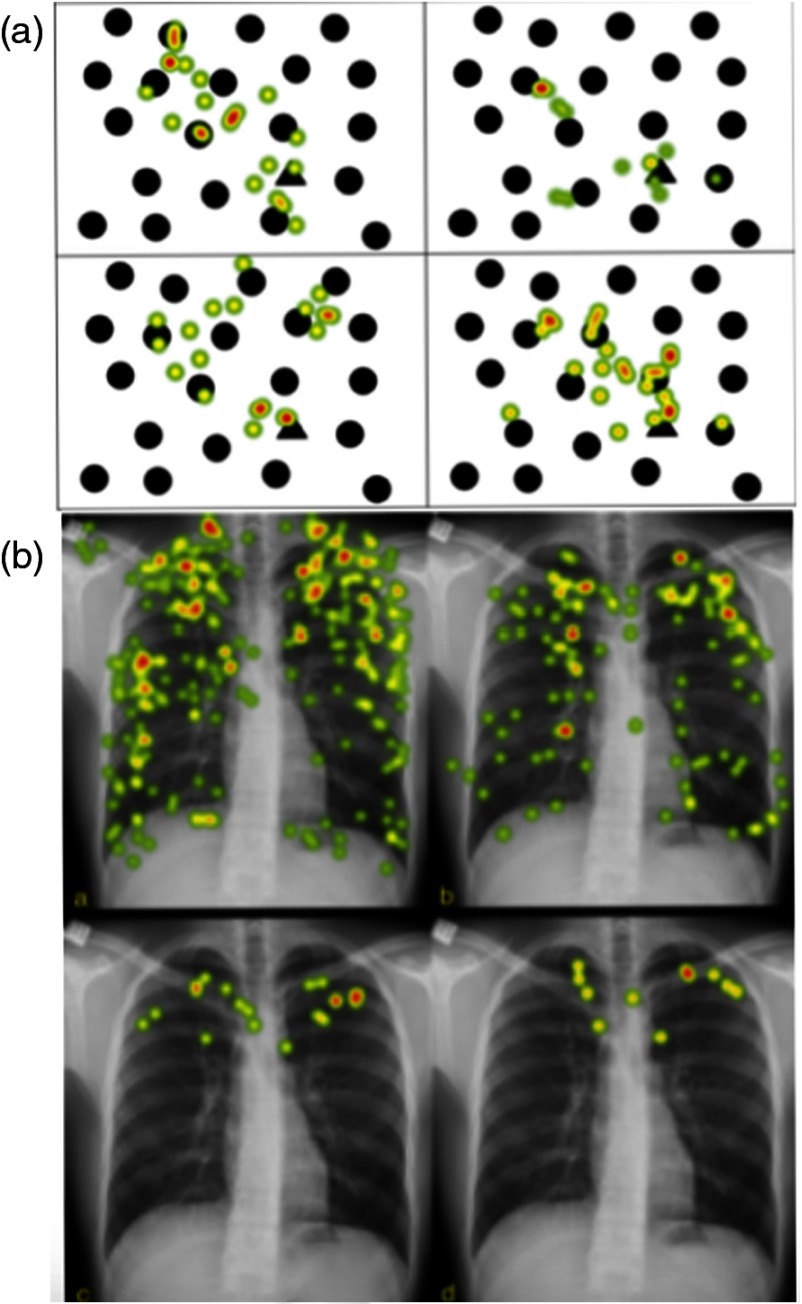
(a) The same image from Fig. 2 with added heat-maps showing participants fixations. Clockwise from the top left shows, in turn, the interns, SHOs, registrars, and consultants fixations while they searched for the triangle. (b) The fixations of the same four groups of physicians reviewing a CXR with a left apical pneumothorax. The trend toward less fixations centered more around the pathology in (b) that is not seen in (a).

Results are shown in Tables 2 and 3 for the complex and simple geometric images, respectively. Results were constant across all metrics with no statistically significant difference either in total or between any experience-adjacent groups. Intergroup variation was also nonsignificant (p>0.1). The aggregate eye-tracking data of participants reviewing the geometric shapes are shown in “heat-map” format [Fig. 3(a)] with Fig. 3(b) showing heat-maps for the same participants looking at a CXR with a left apical pneumothorax. While the changes with experience are evident in the radiologic image, such improvement is not seen in the nonradiologic image.

**Table 2:** Mean eye-tracking data for complex images (range, SD).

	Time to first fixation	Total fixation duration	Max visit count	No. of fixations before ROI
Consultant	6.74 (0.71 to 12.58, 2.65)	1.09 (0.64 to 2.2, 0.57)	2.5 (1 to 6, 0.88)	16.41 (8 to 23, 6.5)
Registrar	6.00 (0.96 to 12.24, 2.99)	0.62 (0.46 to 0.89, 0.21)	1.42 (1 to 3. 0.5)	18.23 (3 to 28, 9.36)
SHO	6.42 (0.4 to 11.09, 3.65)	0.53 (0.24 to 0.87, 0.24)	1.24 (1 to 3. 0.26)	20.08 (1 to 43, 9.36)
Intern	8.49 (0.66 to 18.83, 4.7)	0.65 (0.19 to 2.3, 0.43)	1.93 (1 to 5, 0.85)	22.3 (2 to 60, 15.04)
p value	0.267	0.126	0.951	0.353

**Table 3:** Mean eye-tracking data for simple images (range, SD).

Level and p value	Time to first fixation	Total fixation duration	Max visit count	No. of fixations before ROI
Consultant	0.52 (0.37 to 1.19, 0.15)	0.69 (0.24 to 8.12, 1.24)	2.06 (1 to 5, 1.02)	1.4 (1 to 3, 0.97)
Registrar	0.37 (0.15 to 0.44, 0.03)	0.34 (0.13 to 79, 0.34)	1 (1 to 1,0)	1.61 (1 to 3, 0.75)
SHO	0.37 (0.05 to 0.52, 0.09)	0.64 (0.09 to 2.02, 0.32)	1.8 (1 to 4, 0.83)	1 (1 to 3, 0.67)
Intern	0.42 (0.31 to 0.8, 0.1)	0.35 (0.07 to 76, 0.12)	1 (1 to 1, 0)	1.19 (1 to 2, 0.78)
p value	0.231	0.527	0.066	0.177

### 3.1 Discussion

Image perception has been described in terms of “top down” and “bottom up” theories. Top down theories are those that are global, and bottom up theories are analytical building from small details.^11^ The reproducible ability of experts in radiology to identify pathology far faster than they can fully search the entire image lends evidence to the top down theories.^14^^,^^15^ Top down visual search can, in turn, be thought of in two stages,^16^ stage one using cognitive parallel processing mechanisms to give an overall impression of the image and stage two being a more exhaustive detail oriented process. In radiology, this could mean that interpretation starts with an overview (which often leads to a diagnosis based on initial Gestalt^8^) and, for more complex cases, proceeds to more detailed analysis of the image as a whole. As the participants experience and “bank” of memorized cases grows, the process becomes “guided” and more efficient and accurate.^17^ The bank of experience from which the reader draws also has an effect on perception,^11^ and those with a different frame of reference will actually perceive images differently.

The influence of expertise on the “reading” of a chest radiograph has long been the subject of research. A pioneering paper^18^ explains how the “systematic method” still being taught to medical students and radiology residents today has little bearing on the way an expert examines an image. To examine an image with the highest degree of visual acuity, one needs foveal fixation; however, as stated, most diagnoses are made long before an entire image could be scrutinized with this level of acuity.^14^ While a novice will waste timing moving around an image, the expert will sample “high yield” areas and then focus in on crucial elements to make a diagnosis.

The current research was carried out immediately after an experiment that quantified the four groups of clinician’s diagnostic accuracy and search pattern behavior during identification of pneumothoraces on CXR. That experiment found that not only did diagnostic accuracy and search pattern behavior increase with experience but these two metrics also may reach peak performance at different stages. Previous research has shown that expert radiologist do not outperform lay people in complex nonradiologic search tasks.^1^ Patterns in developing diagnostic accuracy^19^ and visual search^4^ have been identified. The current study aimed to bring these ideas together and try to identify any trends or innate visual search characteristics in groups with different levels of expertise. We found that none of the groups were more successful at identifying targets in complex images or in images that were based on geometric shapes. This is in spite of having significantly different performance in finding a pneumothoraces on chest radiography. The current work also found that when examining participants’ eye-tracking metrics no metrics was statistically different among groups of expertise.

It has been shown that expert radiologists can fixate on an abnormality within 0.25 of a second,^7^ which is much faster than it would take to perform a full foveal search of a given image. The radiologists in our previous work took on average 0.6 s to fixate on a pneumothorax, with interns taking over 2 s.^7^ However, the time to first fixation on any of the targets in the current study was not significantly different (6.74 versus 8.49 s, p=0.267).

In a commentary on the role of image perception research in medical imaging, Manning et al.^3^ summarized the factors that influence the observer in their interpretation as image dependent or image independent. Image-independent factors are mainly cognitive and relate to prior knowledge that the observer has about the image, for example, a consultant knowing a lot more about pathology visible on a CXR than an intern. When readers are asked to view unfamiliar images, this advantage is removed, and image-dependent features, such as how conspicuous a target is from the background, become more important. This “leveling of the playing field” allows us to examine the innate visual search skill of each physician and reveal their baseline ability. The current work demonstrates that this is in fact quite similar. Errors in the current study were all detection rather than decision errors. Previous research has shown that expert errors tend to be decision based rather than detection ones.^20^ This finding held true in the previous work^9^ in which the radiology registrars and consultant radiologists had the same eye-tracking scores but registrars had lower levels of diagnostic accuracy. When nonradiologic images are used to “level the playing field,” the types of errors change from those that an expert might make to those usually made by novices.

There were several limitations to this study. The first was the sample size, both in terms of images and participants. Similar research^1^ has had lower numbers of both images and participants, but we acknowledge that one cannot necessarily generalize conclusions made in one small study to the entire population; intead, this work acts as a proof of concept, and further work is needed. The results can, however, be compared to the CXR experiments with the same number of participants, which did find significant differences in performance among clinician groups of differing expertise. Instead, we employed the expert group (the radiologists) as the gold standard and compared to them as they had already demonstrated their radiologic expertise.

Krupinski^21^ outlines how diagnostic accuracy in observer performance studies is measured using a “figure of merit,” which is usually receiver operator characteristic (ROC) or one of its variants. A free-response ROC (FROC)^22^ analysis would have been preferred for analysis of the maps and targets as it would have allowed for a more thorough analysis of false positives (FPs). However, as the setup was optimized for the initial experiment and the display software did not facilitate switching from ROC to FROC, a simpler form of analysis (number of targets identified) was used. While we can be certain that there were no FPs in the map that did not contain any targets, it is unlikely but possible that there were FPs in the other two images. The number of fixations on ROIs, however, in every case matched the answer given in the MCQs, suggesting that the method was accurate. While this system of analysis is not optimal, it gleaned interesting and consistent data, which, in the opinion of the authors, warrants further investigation.

Zooming and panning (or changing window width or level, but this is not applicable to these kinds of image) were not permitted. While this is different from normal clinical practice, it enables more legitimate interpretation of the eye-tracking data as we can be sure that eye movements are part of visual search as opposed to image optimization.

## 4 Conclusion

Despite clear differences in radiologic expertise, clinician groups showed no difference in nonradiologic search pattern behavior or accuracy across complex images. This implies radiologic expertise is a learned skill and is task specific.
